# Genome-Wide Association Study of Celiac Disease in North America Confirms FRMD4B as New Celiac Locus

**DOI:** 10.1371/journal.pone.0101428

**Published:** 2014-07-07

**Authors:** Chad Garner, Richard Ahn, Yuan Chun Ding, Linda Steele, Samantha Stoven, Peter H. Green, Alessio Fasano, Joseph A. Murray, Susan L. Neuhausen

**Affiliations:** 1 Epidemiology Department, School of Medicine, University of California Irvine, Irvine, California, United States of America; 2 Population Sciences Department, Beckman Research Institute of City of Hope, Duarte, California, United States of America; 3 Celiac Disease Center, Columbia University, New York City, New York, United States of America; 4 Center for Celiac Research, MassGeneral Hospital for Children, Boston, MA, United States of America; 5 Division of Gastroenterology and Hepatology, Department of Immunology, Mayo Clinic, Rochester, MN, United States of America; Tulane University, United States of America

## Abstract

We performed a genome-wide association study (GWAS) of 1550 North American celiac disease cases and 3084 controls. Twelve SNPs, distributed across four regions (3p21.31, 4q27, 6q15, 6q25), were significantly associated with disease (p-value <1.0×10^−7^), and a further seven SNPs, across four additional regions (1q24.3, 10p15.1, 6q22.31, 17q21.32) had suggestive evidence (1.0×10^−7^ < p-value < 1.0×10^−6^). This study replicated a previous suggestive association within *FRMD4B* (3p14.1), confirming it as a celiac disease locus. All four regions with significant associations and two regions with suggestive results (1q24.3, 10p15.1) were known disease loci. The 6q22.31 and 10p11.23 regions were not replicated. A total of 410 SNPs distributed across the eight significant and suggestive regions were tested for association with dermatitis herpetiformis and microscopic colitis. Preliminary, suggestive statistical evidence for association with the two traits was found at chromosomes 3p21.31, 6q15, 6q25, 1q24.3 and 10p11.23, with future studies being required to validate the reported associations.

## Introduction

Celiac disease is predominately a T cell-mediated immune disease caused by sensitivity to the dietary protein gluten. It is primarily a disease of Caucasians, with a population prevalence of approximately 1%[1]–[3]. The role of the major histocompatibility complex (MHC) in celiac disease was first reported 30 years ago [4], [5], with the identification of HLA-DQ2 almost 20 years ago [6]. At least 35% of the disease risk can be attributed to the necessary high-risk HLA types. However, the HLA high-risk genotypes are not sufficient to cause celiac disease. Genome-wide association studies (GWAS) and follow-up studies have identified 40 non-HLA loci that are associated with celiac disease, that combined explain approximately 5% of the disease risk [7]–[11], leaving most of the risk unexplained. In this study of North American celiac disease cases, we conducted a GWAS to identify additional loci and to confirm suspected loci. In addition, at the identified loci, we tested the association of the celiac loci with dermatitis herpetiformis and microscopic colitis. Dermatitis herpetiformis is regarded as an alternative phenotypic expression of gluten-sensitive enteropathy, with little known about the genetic contribution to this phenotype. Microscopic colitis may coexist with celiac disease and is a frequent cause of persistent diarrhea in such patients.

## Materials and Methods

### Subjects

The study included samples contributed by investigators within the North American Celiac Disease Consortium, including Dr. Susan Neuhausen at UC Irvine (now at City of Hope), Dr. Joe Murray at the Mayo Clinic, Dr. Alesio Fasano at the University of Maryland Celiac Disease Center, and Dr. Peter Green at Columbia University. The resources from the four sites were similar in that they used the same serological tests and collected similar questionnaire data. To be considered a celiac case, the individual must have tested serologically positive for IgA tTG and/or IgA EMA and/or had a positive small intestinal biopsy. All of the subjects from the Mayo Clinic and greater than 90% of the subjects from UC Irvine/City of Hope and Columbia University were biopsied. For those with serology only, using sequential testing of IgA ttG followed by IgA EMA testing results in net specificity rates of virtually 100% making it practical to accurately identify those with celiac disease on the basis of serology alone [12], [13].

Celiac patients, recruited at City of Hope and Mayo Clinic, were reviewed for concomitant diagnoses of dermatitis herpetiformis and microscopic colitis. The presence of dermatitis herpetiformis was based on either a confirmatory skin biopsy or clinical documentation of the skin biopsy diagnosis in the medical record that included demonstration of IgA deposits at the dermo-epidermal junction. The presence of microscopic colitis was based on either clinical documentation of the diagnosis in the medical record or confirmatory colonic biopsies showing either lymphocytic or collagenous colitis.

The dbGaP (http://ncbi.nlm.nih.gov/gap/) data repository was used as a source for population controls with GWAS genotype data. Given the prevalence of celiac disease in populations of European descent, the inclusion of population-based sampled controls was expected to result in a negligible reduction in power compared to an equal sized control sample of clinically defined unaffected controls[14]. Data from two studies in dbGaP were utilized because they had large numbers of controls with reported European or Caucasian ancestry that were genotyped using the same Illumina Human 660W Quad v.1A array platform used by CIDR to genotype the celiac disease cases and unaffected controls from the North American Celiac Disease Consortium described above. The NHGRI eMERGE study called “GWAS on Cataract and HLD in the PMRP” contributed genome-wide genotypes, age and sex data for 1343 control samples. The NHGRI GENEVA study called “NHGRI Genome-Wide Association Study of Venous Thrombosis (GWAS of VTE)” contributed genome-wide genotypes, age and sex data for an additional 1295 control samples. Detailed information about both of these studies can be found at the dbGaP website. The genotype data from these 2638 samples from dbGaP were combined with the 530 CIDR-genotyped unaffected controls, giving a total pre-QC control sample size of 3168.

### Ethics Statement

All participants from studies downloaded from dbGaP provided written informed consent under protocols approved by each institution's IRB, as required by dbGaP and posted on the dbGaP website. All participants recruited from University of California Irvine, City of Hope, Columbia University, Mayo Clinic and University of Maryland signed informed consent at the time of study enrollment under protocols approved by each of the respective institutions' IRBs. The subjects from UC Irvine and City of Hope were collected under Dr. Neuhausen's direction with the University of California Irvine IRB approved protocol HS 2002-2521 and the City of Hope/Beckman Research Institute IRB approved protocol 09169. Subjects from the Mayo Clinic were collected under Dr. Murray's direction with the Mayo Clinic IRB approved protocol 1173-99. Subjects from the University of Maryland were collected under the direction of Dr. Fasano with the three University of Maryland IRB approved protocols H-27784, H-29090, and H-29938. The samples from Columbia University were collected under the direction of Dr. Green with the two Columbia University IRB approved protocols 8562 and AAAE8893.

### Genotyping and Quality Control

Center for Inherited Disease Research (CIDR) completed the GWAS genotyping of 1728 celiac disease cases and 530 controls collected in the U.S. (funded by NIH R01 DK081645). The following quality control assessments and corrections were taken prior to the association analysis. All SNPs and samples with less than 98% complete data were excluded from the analysis. SNPs with allele frequencies less than 0.03 or genotype distributions that failed to fit the Hardy-Weinberg equilibrium (HWE) model with a p-value less than 1.0×10^−5^ were filtered from the data. The genotype data were used to test for unknown familial relationships between subjects by analysis of the identity-by-state distribution of the sample, misspecification of sex by analysis of chromosome X/Y genotypes, and population substructure among the subjects by multidimensional scaling of the computed identity-by-state matrix and cluster analysis of the samples. Population genetic outliers identified in the population substructure analysis were excluded from the subsequent association analysis. Exclusions were made when discrepancies could not be resolved. The GenABEL library [15] of R statistical computing environment was used for the quality control assessments.

### SNP Association Analysis

The effect of the SNP genotypes on the dichotomous celiac disease outcome was analyzed using logistic regression models. For the GWAS analysis, the SNPs were coded as continuous variables, with genotypes coded as 0, 1 or 2 to indicate the number of minor alleles in the SNP. The statistical model included the sex of the individual, and the computed principal components of the genomic kinship matrix. There was no significant age effect observed, so the variable was not included in the GWAS analysis. Odds ratios and 95% confidence intervals were calculated from the computed regression coefficients for the SNP genotype effect and standard errors. The dermatitis herpetiformis and microscopic colitis dichotomous phenotypes were analyzed for association using a logistic regression model. All SNPs were analyzed with all samples using a model that included celiac disease status and the SNP as predictors. A second analysis of the two phenotypes included only the celiac disease cases (case-only analysis) and the genotype was the only predictor in the model. The GenABEL library [15] of the R statistical computing environment was used for all association analysis.

## Results and Discussion

A sample of 1728 celiac disease cases and 3168 control individuals were genotyped with the Illumina Human660W-Quad SNP genotyping array for the GWAS. After extensive quality control assessments of the SNPs and samples followed by appropriate filtering, a set of 517,345 SNPs was analyzed in a sample of 1550 cases and 3084 controls. Genome-wide statistical significance was defined as a p-value for the association statistic of less than 1.0×10^−7^. SNPs showing p-values between 1.0×10^−6^ and 1.0×10^−7^ were labeled as having suggestive evidence for association. The SNP association results from the previous celiac disease GWAS and follow-up studies of Dubois et al [7] were used to test for replication of the significant and suggestive findings found in the current GWAS. This replication set included 3,796 cases and 8,154 controls; the samples genotyped on the Hap550 platform described in Dubois et al. [7].

Twelve SNPs met a genome-wide statistical significance threshold of less than 1.0×10^−7^ (Table 1). The SNPs were within the known celiac disease loci on chromosomes 3p21.31, 4q27, 6p15 and 6p25.3, all of which showed significant association in the previous GWAS and follow-up studies by Dubois et al. [7]. These loci were subsequently included in a high-resolution association analysis by Trynka et al. [10] to refine their positions. The locus on chromosome 3p21.31 is intergenic between *CCR3* and *CCR2*. Chromosome 4q27 includes *KIAA1109*, *ADAD1*, *IL2* and *IL21* in a single block of strong linkage disequilibrium. The locus on 6p15 is within intron 2 of the *BACH2* gene, and 6q25.3 includes the 5′ UTR plus 4 kb of the *TAGAP* gene. Identification of these sites is consistent with and confirms previous findings as to their locations and odds ratios.

**Table 1:** GWAS and replication results for SNPs showing genome-wide significant and suggestive evidence for association.

			GWAS Results			Dubois et al. Results	
SNP	Position	Freq.	P-value	O.R.	95% C.I.	P-value	O.R.
**Regions with Significant Evidence for Association (p-value ≤1.0×10^−7^)**							
**3p21.31** Intergenic region between *CCR3* and *CCR2*							
rs13096142	46,256,748	0.30	4.44×10^−8^	1.30	1.18–1.43	7.96×10^−7^	1.16
rs11711054	46,320,615	0.32	8.80×10^−8^	1.29	1.17–1.41	1.50×10^−6^	1.16
rs6441961	46,327,388	0.32	6.63×10^−8^	1.29	1.19–1.42	6.32×10^−7^	1.16
**4p27** Includes *KIAA1109, ADAD1, IL2, IL21*							
rs1997179	123,204,083	0.14	4.01×10^−9^	0.67	0.59–0.77	1.28×10^−9^	0.77
rs13132933	123,230,037	0.16	1.02×10^−9^	0.68	0.60–0.77	7.85×10^−12^	0.76
rs11938795	123,292,459	0.24	9.56×10^−7^	0.77	0.69–0.85	2.07×10^−8^	0.82
rs13151961	123,334,952	0.16	2.59×10^−11^	0.65	0.57–0.73	1.29×10^−13^	0.73
rs11734090	123,447,563	0.24	6.16×10^−7^	0.76	0.69–0.85	1.53×10^−8^	0.82
rs6851362	123,482,896	0.24	2.01×10^−7^	0.75	0.68–0.84	4.10×10^−8^	0.83
rs6840978	123,774,157	0.18	7.92×10^−10^	0.69	0.61–0.78	3.95×10^−11^	0.77
**6q15** Intron 2 of *BACH2*							
rs2474619	90,936,756	0.38	2.39×10^−8^	0.78	0.71–0.85	0.00037	0.90
rs1394220	91,076,025	0.44	5.30×10^−7^	1.25	1.14–1.36	0.00018	1.11
**6q25.3** 4kb 5′ and 5′ UTR of *TAGAP*							
rs1738074	159,385,965	0.43	3.40×10^−8^	1.28	1.18–1.40	4.79×10^−7^	1.15
rs212402	159,392,283	0.35	8.00×10^−9^	1.31	1.20–1.44	1.01×10^−6^	1.16
rs169858	159,404,764	0.30	1.27×10^−7^	1.29	1.17–1.41	0.031	1.07
rs212388	159,410,424	0.41	1.82×10^−8^	1.29	1.19–1.41	4.13×10^−6^	1.14
rs654690	159,434,766	0.34	3.05×10^−8^	1.30	1.18–1.42	1.97×10^−5^	1.14
rs2249937	159,435,297	0.32	2.74×10^−7^	1.28	1.16–1.40	5.26×10^−5^	1.13
**Regions with Suggestive Evidence for Association (1.0×10^−7^ ≤ p-value ≤1.0×10^−6^)**							
**1q24.3** Includes *FASLG, TNFSF18, TNFSF4*							
rs9286879	171,128,857	0.25	8.25×10^−7^	1.29	1.17–1.42	0.24	1.04
rs2157453	171,130,571	0.25	7.65×10^−7^	1.29	1.17–1.42	0.21	1.04
**6q22.31** *NKAIN2*							
rs531930	124,785,206	0.22	5.32×10^−7^	1.30	1.17–1.44	0.64	0.98
**10p15.1** Intergenic between *PFKFB3* and *PRKCQ*							
rs4558075	6,441,631	0.18	2.01×10^−7^	0.73	0.65–0.82	0.013	0.84
rs10796045	6,442,747	0.18	4.63×10^−7^	0.74	0.66–0.83	0.014	0.85
**17q21.32** *HOXB9*							
Rs8081319	46,256,748	0.13	8.36×10^−7^	1.36	1.20–1.54	0.47	0.97

Four regions showed suggestive evidence for association with celiac disease, with these regions having at least one SNP meeting suggestive evidence threshold and no SNPs showing significant association (Table 1). Two SNPs on 10p15.1, rs4558075 and rs10796045, provided suggestive evidence to the region, with the SNPs having p-values of 0.013 and 0.014, respectively, for replication. The analysis of the region carried out by Trynka et al. [10] provided significant evidence for a celiac disease locus at 10p15.1. On chromosome 1q24.3, two SNPs in strong linkage disequilibrium (LD) showed suggestive evidence for association with celiac disease; however the SNPs showed no evidence for association, or replication, in the Dubois et al. [7] study. In the Dubois study, SNP rs859637 at 1q24.3 position 170,974,795 showed suggestive evidence for association, with GWAS and follow-up study p-values of 8.15×10^−5^ and 5.68×10^−3^, respectively. Subsequent analysis of the region by Trynka et al. [10] identified two significant association signals across the approximately 300 kb region. The two SNPs shown in Table 1 (rs9286879 and rs2157453) mark one of the two signals reported by Trynka et al. [10]. SNP rs859665 at position 170,931,065 on 1q24.3 showed a p-value of 4.48×10^−4^ in the GWAS and is in very weak linkage disequilibrium with the two SNPs in the same region shown in Table 1 (*D*' = -0.404 and *R* ^2^ = 0.025 between rs2157453 and rs859665). The SNPs rs859665 and rs2157453 showed significant independent effects in a logistic regression model that included both SNPs. The lack of replication observed for the two 1q24.3 SNPs shown in Table 1 can likely be attributed to sampling variation and statistical chance. SNPs rs531930 and rs8081391 on chromosomes 6q22.31 and 17q21.32, respectively, showed no evidence for association in the Dubois et al. [7] study, indicating that these suggestive results were most likely due to statistical chance and are not marking a real celiac disease region.

**Figure 1:**
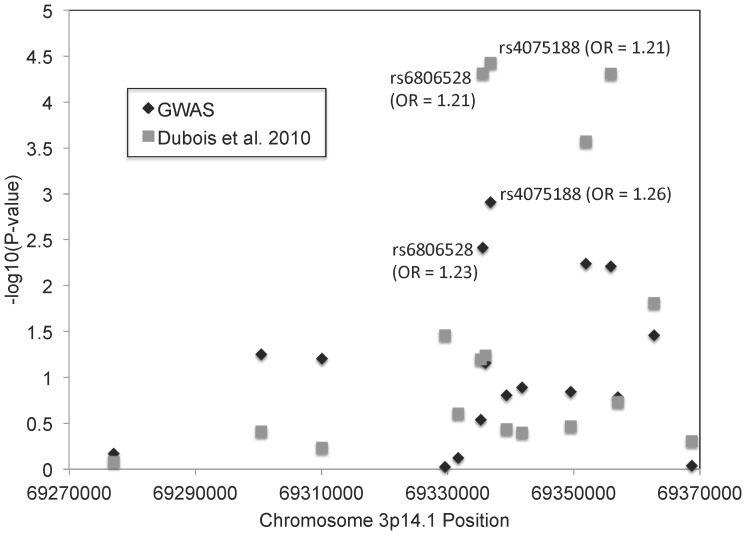
Association results for 17 SNPs from the current GWAS and the GWAS reported by Dubois et al. (REF) within the FRMD4B gene on 3p14.1.

Dubois et al [7] reported 13 SNPs in as many regions as showing suggestive statistical evidence for association with celiac disease; all 13 SNPs were genotyped and analyzed in the current GWAS. Five of the suggestive SNP associations were subsequently replicated by Trynka et al. [10] and are among the 40 known celiac disease regions. We investigated the association results in the current GWAS at these eight suggestive SNP loci that have not been subsequently replicated to determine if there was evidence for association in this North American study. Of the eight previously suggestive but unreplicated SNPs, only SNP rs6806528 on chromosome 3p14.1 showed evidence for association in the current GWAS. The associated region spans a 100 kb LD block. The most significant association identified in this GWAS was at rs4075188 with a p-value of 0.0012; the same SNP showed a p-value of 3.75×10^−5^ in the Dubois et al. [7] study and was the most significantly associated SNP in the region in that study. Figure 1 shows the results for all 17 SNPs across the LD block that were analyzed in the current study and by Dubois et al. [7]. The greater statistical significance reported by Dubois et al. [7] is a reflection of the larger sample size. The Pearson correlation between the log-transformed p-values of the two studies is 0.85 (p-value  = 1.33×10^−5^). Under a null hypothesis of no association and independence of the two samples, the very high correlation between the results of the two studies is highly improbable. Furthermore, the odds ratio estimates from the current and the Dubois et al. [7] GWAS were consistent across the SNPs showing association. The LD block lies completely within the FRMD4B gene. *FRMD4B* is ubiquitously expressed and its product is believed to be involved in the establishment of epithelial cell polarity, and may also function as a scaffolding protein.

There were 410 SNPs genotyped within the eight LD blocks containing the celiac associated regions shown in Table 1. These 410 SNPs were tested for association with the celiac-related conditions dermatitis herpetiformis (DH) and microscopic colitis (MC). Of the 1550 celiac disease cases in the study, 59 (3.8%) and 69 (4.5%) were positive for DH and MC, respectively. For DH, most studies suggest that the prevalence of DH in celiac disease is between 5 and 10%.[2] For MC, the prevalence in this study is consistent with previous reports of 4.3% of celiac patients having concomitant MC.[16] Microscopic colitis includes lymphocytic colitis (LC) and collagenous colitis (CC). The association between MC and celiac disease is well recognized, as both are associated with HLA-DQ2.[17], [18] For the case-control analysis, the genotype distributions of the 59 and 69 respective cases were compared against all celiac cases without these conditions and the controls, while adjusting for celiac disease status. The inclusion of the non-phenotyped dbGaP controls was not expected to have a detrimental effect on the statistical power given the rarity of the conditions. The 17 SNPs were subsequently tested using only the celiac disease cases in order to ensure that the results were not due to residual confounding with celiac disease status. Table 2 shows the association test results for the 17 SNPs that had p-values less than 0.01 in the case-control analysis. None of the 410 tested SNPs met a Bonferroni multiple test corrected p-value threshold of less than 0.00012 (0.05/410).

**Table 2:** Results of association analysis of microscopic colitis and dermatitis herpetiformis across celiac associated regions.

			Case-Control			Case-only
SNP	Position	Freq.	P-value	O.R.	95% C.I.	P-value
**Microscopic Colitis**						
**Chromosome 3p21.31**: 45,904,804–46,625,997						
rs4683148	45,931,064	0.39	0.0044	1.66	1.17–2.35	0.0097
rs1072755	45,950,987	0.39	0.0034	1.68	1.19–2.37	0.0076
rs4535265	45,952,870	0.39	0.0037	1.68	1.18–2.38	0.013
rs2234358	45,964,048	0.49	0.0021	0.57	0.40–0.82	0.0019
rs3796375	45,983,794	0.42	0.0041	1.67	1.18–2.36	0.011
rs737452	46,002,414	0.42	0.0039	1.67	1.18–2.36	0.016
rs2373154	46,146,102	0.43	0.0045	1.66	1.17–2.35	0.0088
**Chromosome 6q15**: 90,863,556–91,096,529						
rs207270	90,885,603	0.46	0.0095	1.59	1.12–2.27	0.011
rs4142967	91,053,070	0.46	0.0023	1.72	1.21–2.43	0.00044
rs12212193	91,053,490	0.46	0.0023	1.72	1.21–2.43	0.00044
rs285640	91,085,518	0.33	0.00058	0.45	0.29–0.71	0.0009
rs1847473	91,093,744	0.27	0.0018	1.74	1.23–2.46	0.0012
**Chromosome 6q25.3**: 159,242,314–159,461,818						
rs1738074	159,385,965	0.43	0.00044	0.52	0.36–0.75	0.0041
rs2451241	159,441,116	0.43	0.0088	1.58	1.12–2.24	0.046
**Chromosome 1q24.3**: 170,917,308–171,207,073						
rs2227203	171,145,646	0.46	0.0096	0.62	0.43–0.89	0.036
						
**Dermatitis Herpetiformis**						
**Chromosome 1q24.3**: 170,917,308–171,207,073						
rs10798176	170,942,148	0.16	0.00076	2.15	1.38–3.35	0.00025
**Chromosome 10p11.23**: 30,004,000–30,041,000						
rs11007696	30,012,556	0.32	0.0052	0.51	0.32–0.82	0.0044

SNPs on chromosomes 3p21.31, 6q15, 6q25.3 and 1q24.3 showed preliminary, suggestive evidence for association with MC (uncorrected p-value <0.01), with the smallest p-values observed at chromosomes 6q15 (rs285640 p-value  = 0.00058) and 6q25.3 (rs1738074 p-value  = 0.00044). The 6q15 region is located within an intron of *BACH2*. *BACH2* codes for a transcription regulator protein with a role in the regulation of B cells. In addition to its known association with celiac disease, it is also associated with ulcerative colitis, Crohn's disease, and Type I diabetes mellitus.[19] At 6q25.3, the SNP rs1730874 was among the celiac associated SNPs shown in Table 1 and it is located in the five prime untranslated region (5′ UTR) of *TAGAP*, a gene coding for a GTPase-activating protein that is involved in T-cell activation. This SNP, in addition to its association with celiac disease, is associated with ulcerative colitis, particularly in ulcerative colitis patients with a family history of the disease.[20]

At 3p21.31, SNP rs2234358 is located in the three prime untranslated region (3′ UTR) of *CXCR6*, a chemokine receptor on T cells. *CXCR6* has been associated with other colitides, particularly Crohn's disease. CYCL16, a ligand of CXCR6 has been shown to be upregulated in colons of mouse models of colitis[21] and increased CYCL16 serum levels have been observed in patients with inflammatory bowel disease.[22] Also in this region, SNPs rs1072755, rs4535265, rs3796375, rs737452 are intronic variants in *FYCO1*. *FYCO1* codes for a protein involved in autophagosome trafficking and has been associated with congenital cataracts.[23] *FYCO1* has not been previously associated with colitides such as microscopic colitis; however variants in other autophagy genes have been implicated in Crohn's disease.[24], [25]

One SNP each on chromosomes 1q24.3 and 10p11.23 showed preliminary, suggestive evidence for association with DH. At 1q24.3, SNPs rs2227203 and rs10798176 were associated with MC and DH, respectively.

DH is an extraintestinal manifestation of celiac disease, with virtually all patients carrying either HLA-DQ2 or DQ8 haplotypes[26]. There are no known reasons for why DH manifests in some celiac disease cases and not others. It may be that loci at 1q24.3 and 10p11.23 provide a permissive condition for the development of the cutaneous disease, however, it is most probable that the strongest genetic determinants of DH are not celiac disease loci and will require a large, sufficiently powered GWAS of the disease to be discovered.

Although the HLA genotypes are necessary for celiac disease, they are common and insufficient to cause disease, and each individual celiac patient will have a genetic etiology that includes non-HLA disease alleles. Considerable progress has been made towards elucidating the celiac loci with common disease alleles. This report adds support to previously identified loci, and provides statistical evidence for a celiac disease locus in the *FRMD4B* gene on 3p14.1, providing the needed confirmation that it is a celiac disease locus. This now brings the total number of identified loci for celiac disease to 41. Furthermore, we found preliminary, suggestive associations of MC and DH with celiac-associated loci at chromosomes 3p21.31, 6q15, 6q25.3,1q24.3, and 10p11.23. Further studies are needed to validate these findings.
